# The role of Ubiquitination in Apoptosis and Necroptosis

**DOI:** 10.1038/s41418-021-00922-9

**Published:** 2021-12-15

**Authors:** Jamie Z. Roberts, Nyree Crawford, Daniel B. Longley

**Affiliations:** 1 The Patrick G Johnston Centre for Cancer Research, Queen’s University Belfast, Belfast, UK; 2 Almac Discovery Laboratories, Health Sciences Building, Queen’s University Belfast, Belfast, UK

**Keywords:** Protein-protein interaction networks, Deubiquitylating enzymes, Ubiquitin ligases, Ubiquitylation

## Abstract

Cell death pathways have evolved to maintain tissue homoeostasis and eliminate potentially harmful cells from within an organism, such as cells with damaged DNA that could lead to cancer. Apoptosis, known to eliminate cells in a predominantly non-inflammatory manner, is controlled by two main branches, the intrinsic and extrinsic apoptotic pathways. While the intrinsic pathway is regulated by the Bcl-2 family members, the extrinsic pathway is controlled by the Death receptors, members of the tumour necrosis factor (TNF) receptor superfamily. Death receptors can also activate a pro-inflammatory type of cell death, necroptosis, when Caspase-8 is inhibited. Apoptotic pathways are known to be tightly regulated by post-translational modifications, especially by ubiquitination. This review discusses research on ubiquitination-mediated regulation of apoptotic signalling. Additionally, the emerging importance of ubiquitination in regulating necroptosis is discussed.

## Facts

- The intrinsic apoptotic pathway is predominantly regulated by degradative types of ubiquitination targeting both anti- (e.g. Mcl-1) and pro- (e.g. Bim) apoptotic proteins.
- In the extrinsic apoptotic pathway, the core apoptosis promoting proteins (FADD and Caspase-8) are predominantly modified with non-degradative types of ubiquitin chains, which mostly inhibit their pro-apoptotic functions.
- FLIP levels regulate Caspase-8 activity, and ubiquitination of FLIP seems to exclusively affect its stability.
- RIPK1, RIPK3 and MLKL are specifically ubiquitinated during TNF-α-induced necroptosis.
- During necroptosis, RIPK1 ubiquitination can either promote or inhibit necroptosis, while RIPK3 ubiquitination predominantly inhibits necroptosis.

## Open questions

- How do non-degradative types of FADD and Caspase-8 ubiquitination inhibit Caspase-8 activity?
- What is the exact spatial and temporal order of protein recruitment and ubiquitination events in TRAIL-R signalling and how similar are these to TNFR1 signalling?
- How do non-degradative ubiquitin chains promote/reduce necroptosis?

## Introduction

Cell death is crucial for embryogenesis and maintaining homoeostasis within the body. Programmed cell death is a term used to classify a range of innate signalling pathways such as apoptosis, necroptosis, pyroptosis and ferroptosis that result in the termination of a cell. Dysfunctional cell death is a hallmark of cancer and is also known to cause neurodegenerative and immune-related diseases; hence, it is important to understand the regulation of these pathways. It is apparent that ubiquitination is intimately involved in the regulation of multiple cell death pathways. The extrinsic apoptotic pathway is a good example of how extensive, complex and important ubiquitination can be in regulating a signalling pathway [1]. In this review, we will first discuss the molecular biology of ubiquitination and then we will explore the intrinsic and extrinsic apoptotic pathways as well as the necroptotic pathway.

### The complexities of ubiquitination

Ubiquitination is a type of post-translational modification that involves the covalent attachment of ubiquitin (Ub) to a target protein. Ub conjugation is reliant on an E1 (ubiquitin-activating enzyme), E2 (ubiquitin-conjugating enzyme) and E3 (ubiquitin ligase) enzyme [2, 3], and results in the formation of an isopeptide bond between the C-terminus of Ub (Gly76) and, most commonly, the side chain of a lysine amino acid in the target protein [3].

#### The ubiquitination and deubiquitination machinery

The specificity of Ub conjugation system is reflected in the number of each type of enzyme present, with two E1 activating enzymes, 38 E2 conjugating enzymes and ~600–1000 E3 ligases encoded by the human genome [4]. The E3 ligases give specificity to the conjugation process since they directly bind to the substrate [3]. Traditionally, E3 ligases are classified into three families: the Homology to E6-AP C Terminus, Really Interesting New Gene (RING) and RING-in-between-RING families [5–7]. Recently, two new families of E3 ligases have been classified, named the RING-Cys-Relay and RNF213-ZNFX1 families [8, 9].

Like many post-translational modifications, ubiquitination can be reversed; this is carried out by the DeUBiquitinating enzyme (DUB) family. Generally, it is thought that DUB specificity is aimed towards different kinds of Ub chain conjugates [10] (which will be discussed further below). However, DUBs are continuously being shown to associate with complexes containing E3 ligases, negatively regulating their activity [11]; therefore, understanding what complexes DUBs interact with will also allow us to understand their physiological specificity.

#### Monoubiquitination: ubiquitination in its simplest form

**Fig. 1:**
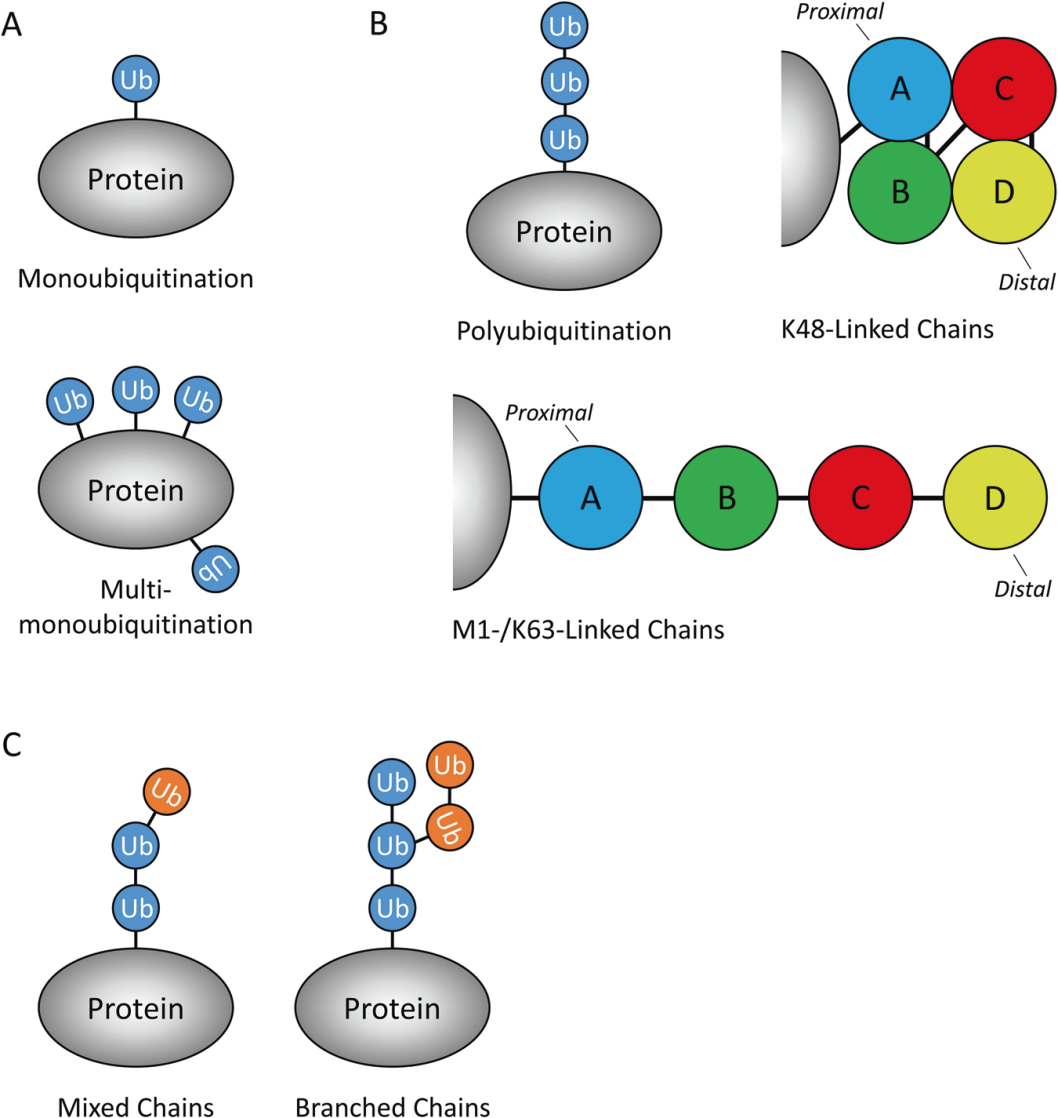
The complexities of ubiquitination. **A** Diagram of monoubiquitination and multi-monoubiquitination. **B** Simplistic representations of K48-linked and M1/K63-linked polyubiquitination (tetraubiquitin) chains. A (blue), B (green), C (red) and D (yellow) represent Ub monomers within a tetraubiquitin chain, with A being proximal and D being the distal Ub monomers. **C** Simplistic representations of mixed/branched Ub chains; blue and orange circles represent Ub monomers within a Ub chain, the colour indicates which residue (unspecified) the Ub monomer is conjugated to the previous Ub monomer (e.g. K48, K63, M1).

A single Ub molecule can be attached to a protein (monoubiquitination), or multiple Ub monomers can be attached simultaneously to several different lysines in a protein (multi-monoubiquitination) (Fig. 1A). Monoubiquitination has been shown to regulate trafficking of important signalling molecules, including H- and N-RAS [12], EGFR [13], HDAC7 [14] and TRAF4 [15], in addition to regulating protein complex formation [16].

#### Polyubiquitination: chain architecture

Ub monomers can form more complex structures creating ‘chains’ of Ub attached to Ub’s lysine residues (Fig. 1B). When this occurs, it is termed polyubiquitination and can have diverse signalling outcomes depending on the type of chain structure formed. Ub itself contains 7 lysines (K6, K11, K27, K29, K33, K48, K63) and an N-terminal methionine (M1); all of these residues can be conjugated to additional Ub molecules and, when a specific residue ‘links’ all the Ub monomers together, the chain is named after that residue, such as K48-linked or K63-linked chains. The Ub residue used for polyubiquitination dictates the structure of the Ub chain; M1- and K63-linked chains adopt an ‘open’ conformation that resembles a linear chain, while K48-linked chains have a compact ‘zig-zag’ globular conformation [17–19] (Fig. 1B). It should be noted that in the literature, M1-linked chains are commonly referred to as ‘linear chains’ and this does not refer to K63-linked chains. The structure of the different chain conjugates partially reflects their functions; for example, K63- and M1-linked chains are known for their roles in recruitment of protein complexes, while K48-linked chains are known to target proteins for degradation by the ubiquitin proteasome system (UPS).

#### Polyubiquitination: mixed and branched chains

Different types of innate immune signalling, TNFR1 signalling included, have been shown to modify RIPK1/2 with K63-linked chains, which are then further elongated with M1-linked chains, to generate ‘mixed’ chains [20] (Fig. 1C). This is important for the juxtaposition of the Transforming growth factor-β-Activated Kinase 1 (TAK1) and IκB Kinase (IKK) complexes, which are necessary for efficient NF-κB signalling, because each complex preferentially binds to either K63- or M1-linked chains, respectively. Furthermore, their co-localisation allows the TAK1 complex to phosphorylate and activate the IKK complex, thereby propagating NF-κB signalling [21].

Additionally, a ‘branched’ version of mixed chains was recently reported [22] (Fig. 1C). Standard techniques, using mass spectrometry, are ‘blind’ to detection of branched chains; however, Ohtake et al. developed a method to detect and quantify K48/K63 branched chains [22]. Additionally, the same study reported that the typically non-degradative K63-linked chains could be modified with K48-linked branched chains and induce degradation [22]. The study of mixed and branched chains is still in its early stages, but clearly adds an important new layer of complexity to protein regulation by ubiquitination.

### The intrinsic apoptotic pathway

**Fig. 2:**
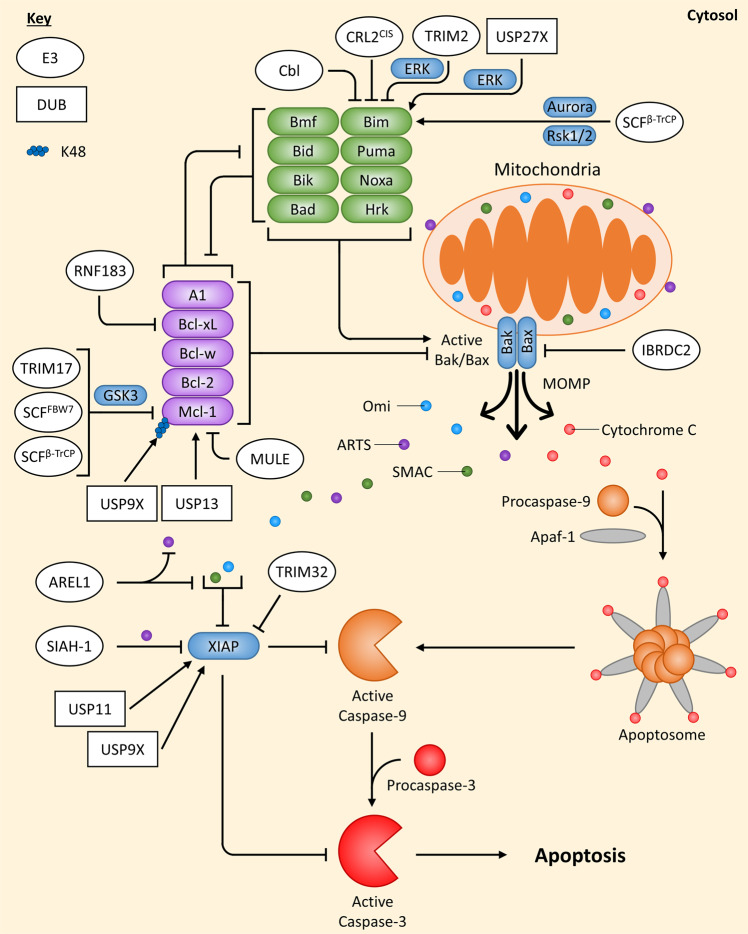
Overview of ubiquitin-mediated regulation of the intrinsic apoptotic pathway. Cellular stress signals that induce intrinsic damage to the cell, such as DNA damage, affect the levels of the Bcl-2 family of proteins. Depending on the levels of pro-apoptotic (green) and anti-apoptotic (purple) Bcl-2 family proteins this can allow active Bak/Bax to induce MOMP. Cytochrome C is released from the mitochondria and can form the apoptosome with Apaf-1 and Procaspase-9, which leads to active Caspase-3/7/9 generation and apoptosis induction. However, XIAP is a potent inhibitor Caspase-3/7/9, but is neutralised by IAP antagonists (SMAC, Omi, ARTS) when they are released from the mitochondria as well. Regulation of the intrinsic apoptotic pathway by E3 ligases (white ovals) and deubiquitinating enzymes DUBs (white rectangles) are indicated. If an E3 ligase/DUB is pointing at a specific Ub chain (indicated by a chain of coloured circles), this means the E3 ligase/DUB is known to regulate the protein by targeting this specific chain; E3 ligases always add Ub chains while DUBs always remove Ub chains. Arrows always indicate positive regulation of a protein they point at (or the protein at the end of the Ub chain they point at), while flat-headed lines indicate the opposite.

Intrinsic apoptosis senses a wide range of internal stress signals that are usually produced by cellular stresses, such as DNA damage, high levels of reactive oxygen species, endoplasmic reticulum (ER) stress or nutrient starvation. All types of intracellular stress signals eventually converge at the mitochondria where the fate of the cell is decided. Members of the Bcl-2 family can be both pro- and anti-apoptotic, and a balance between their levels determines whether apoptosis occurs or not. There are three subclasses of the Bcl-2 family: the pro-apoptotic executioners, the pro-apoptotic BH3-only proteins and the anti-apoptotic Bcl-2 proteins. Since the balance between pro- and anti-apoptotic Bcl-2 family members’ levels is key to determining cell fate, it is unsurprising that ubiquitin-mediated degradation regulates this pathway in several ways (Fig. 2).

#### The Bcl-2 family executioners

Bax and Bak are the main pro-apoptotic executioners that can oligomerise in the outer mitochondrial membrane, when activated, forming pores that induce Mitochondrial Outer Membrane Permeabilization (MOMP). The E3 ligase IBRDC2 has been reported to target Bax for ubiquitin-mediated degradation and, interestingly, IBRDC2 localises to the mitochondria only when active Bax is localised there, suggesting that IBRDC2 plays a role in stopping MOMP induced by active Bax [23].

#### The BH3-only proteins

The pro-apoptotic BH3-only proteins (Bid, Bim, Puma, Noxa, Bad, Bmf, Hrk and Bik) induce Bax/Bak pore formation, either by directly activating Bax/Bak or passively by sequestering the anti-apoptotic Bcl-2 proteins [24, 25]. Only Bim has been reported to be regulated by several E3 ligases/DUBs, all via degradation. The extra-long splice variant of Bim (BimEL) has been shown to be marked for degradation by the E3 ligase Cbl [26]; however, another study showed this not to be the case in different cell lines [27], suggesting that Cbl’s effects on Bim might be cell type-specific. Additionally, BimEL was shown to be marked for degradation by the E3 ligase complex CRL2^CIS^, which contains Elongin B/C, Cullin-2 and CIS [28]. Phosphorylation of Bim by ERK in response to ischaemia was shown to induce an interaction between Bim and the E3 ligase TRIM2, resulting in its ubiquitination and degradation [29]. Additionally, Rsk1/2 [30] and Aurora kinase [31] were shown to phosphorylate BimEL at Ser93/Ser94/Ser98, inducing its ubiquitination and degradation via the SCF^β-TrCP^ complex. The DUB USP27X can interact with and stabilise Bim by reducing its ubiquitination levels, but paradoxically, this was also reported to be dependent on ERK-mediated phosphorylation of Bim [32]. This could be explained by the presence of E3 ligases and DUBs within the same complexes [11].

#### The anti-apoptotic Bcl-2 proteins

The anti-apoptotic Bcl-2 proteins (Bcl-2, Bcl-xL, Bcl-w, Mcl-1 and A1) can inhibit apoptosis either by the direct binding and inhibition of Bax/Bak or by sequestering BH3-only proteins that directly interact with Bax/Bak [33]. Several studies have shown that Mcl-1 is regulated by degradative types of ubiquitination. The DUB USP9X was reported to stabilise Mcl-1 by removing K48-linked chains from it; however, while K48-linked (but not K63-linked) chains could be detected on tagged Mcl-1 with a linkage specific antibody, USP9X was never demonstrated to specifically remove this linkage type from Mcl-1 [34]. Another DUB, USP13, was also shown to stabilise Mcl-1 by reducing its ubiquitination [35].

In neuronal cells, the E3 ligase TRIM17 was shown to ubiquitinate and degrade Mcl-1 in a process dependent on Mcl-1’s phosphorylation by GSK3 [36]. The E3 ligase MULE was also shown to interact with Mcl-1, resulting in its ubiquitination and degradation [37]; however, basal levels of Mcl-1 were not affected in MULE-deficient cells [38]. Interestingly, it was found that the BH3-only proteins Bim and Puma could displace the MULE:Mcl-1 interaction [39–41], whereas Noxa’s interaction with Mcl-1 increased the MULE:Mcl-1 interaction, triggering its degradation, while also reducing the DUB USP9X’s association with MULE [40, 42]. The SCF^β-TrCP^ and SCF^FBW7^ E3 ligase complexes have both been implicated in regulating the intrinsic apoptotic pathway via Mcl-1 ubiquitination and degradation. GSK3 was observed to phosphorylate Mcl-1 at Ser159/Thr163 which induced both β-TrCP’s and FBW7’s association with Mcl-1, resulting in its ubiquitination and degradation [43]. Additionally, phosphorylation of Mcl-1 at Thr92 by CDK1-cyclin B1, in response to mitotic arrest, was shown to induce ubiquitination and degradation of Mcl-1 by the APC/C(Cdc20) E3 ligase complex [44].

Apart from Mcl-1, a recent study demonstrated that Bcl-xL is also regulated by Ub-mediated degradation, by the transmembrane E3 ligase RNF183 in response to ER stress [45]. Although RNF183 is usually localised to the ER and Bcl-xL to the mitochondria, the mitochondria and ER frequently come into close proximity and form membrane contact sites [46]. Additionally, a substrate on the mitochondria has been reported to be efficiently ubiquitinated by an ER-bound E3 ligase [47], indicating the plausibility of ER-located RNF183 being able to ubiquitinate mitochondria-located Bcl-xL.

#### Downstream of MOMP

Once active Bak/Bax has induced MOMP, pro-apoptotic factors such as cytochrome c and IAP antagonists (SMAC, Omi, ARTS) are released from the mitochondria into the cytosol. In the presence of ATP; cytochrome c, Apaf-1 and Procaspase-9 interact and oligomerise, forming a complex that is known as the apoptosome. The apoptosome allows Procaspase-9 to form homodimers, which induces its enzymatic activity and autoproteolytic cleavage, resulting in its activation. Active Caspase-9 can then cleave the executioner Caspases, Procaspase-3/7, thereby promoting apoptosis. However, if the anti-apoptotic protein XIAP is present, it can potently block Caspase-3/7/9 activity [48].

XIAP itself is an E3 ligase and a potent inhibitor of both the intrinsic and extrinsic apoptotic pathways. Its E3 ligase activity does not seem necessary for its function in inhibiting apoptosis in humans [49] since its primary method of inhibiting apoptosis is by directly binding to Caspase-3/7/9 [50]. Though, the E3 ligase activity of XIAP is important for its auto-ubiquitination, which results in its degradation [51]. Recently the E3 ligase TRIM32 was demonstrated to either stimulate XIAP’s auto-ubiquitination or directly ubiquitinate it, resulting in its degradation [52], while the DUB USP11 was identified to interact with the BIR2 domain of XIAP and induce XIAP’s deubiquitination and stabilisation [53]. Furthermore, the DUB USP9X has been suggested to deubiquitinate and stabilise XIAP to promote cell survival during the mitotic spindle assembly checkpoint [54]. Additionally, XIAP itself has been reported to be an E3 ligase for several apoptotic-related proteins including Bcl-2, SMAC, AIF and ARTS [55–58].

To overcome XIAP, the IAP antagonists, SMAC (Diablo), Omi (HtA2) and ARTS (Sept4_i2), are released from the inner mitochondrial membrane space following MOMP or, in the case for ARTS, from the outer mitochondrial membrane preceding MOMP [48] and inhibit XIAP [59–61]. Inhibition of XIAP by IAP antagonists is conducted in a few ways but, in particular interest, ARTS is able to do so by activating XIAP auto-ubiquitination and/or ubiquitination through the E3 ligase SIAH-1 [61]. The E3 ligase AREL1 was reported to ubiquitinate and degrade SMAC, Omi and ARTS under apoptotic conditions, likely due to AREL1 being localised in the cytosol and therefore only in proximity to XIAP antagonists following MOMP [62].

### The extrinsic apoptotic pathway

The extrinsic pathway of apoptosis senses extracellular apoptotic signals through Death Receptors that are present as transmembrane receptors in the plasma membrane. These receptors include TNFR1 and TRAIL-R1/2 (DR4/5), and are activated by their corresponding ligands TNF-α and TRAIL, respectively. In many normal cells, Death Receptors primarily activate pro-survival and inflammatory pathways via activation of the MAPK and NF-κB pathways [63, 64]; however, in cancer cells, activation of the death receptors typically induces apoptosis. Since a detailed review surrounding the ubiquitination events in extrinsic apoptosis is already present [1], this review will discuss the latest discoveries and add to what is already known.

#### TNFR1-induced complexes

**Fig. 3:**
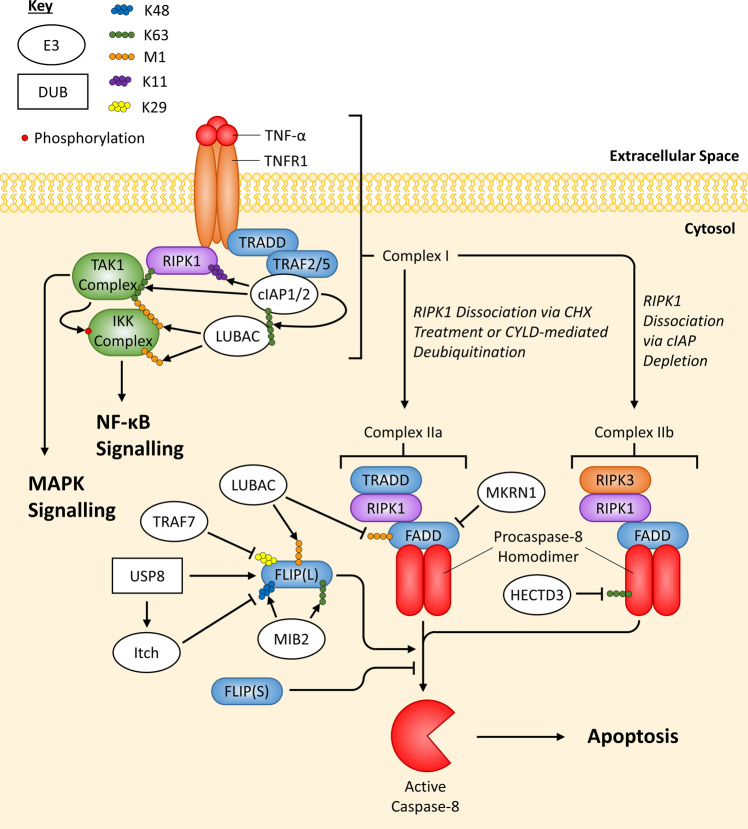
Overview of ubiquitin-mediated regulation of TNFR1 apoptotic signalling. When trimeric TNF-α binds to trimeric TNFR1, Complex I is formed through the recruitment of proteins via protein:protein interactions and interactions mediated by Ub chains, leading to activation of the NF-κB and MAPK pathways. The basic ubiquitination events of Complex I have been displayed since this is not the focus of this review, but a more detailed review of the ubiquitination events occurring at Complex I can be found here [124]. If RIPK1 is not ubiquitinated then it can dissociate from Complex I and form Complex IIa or IIb. Complex IIa/b can generate active Caspase-8, which cleaves Procaspase-3 and induces apoptosis. E3 ligases/DUBs that regulate Complex IIa/b do not necessarily regulate each complex specifically, it is for convenience they have been drawn this way. Regulation by E3 ligases (white ovals) and deubiquitinating enzymes DUBs (white rectangles) are indicated. If an E3 ligase/DUB is pointing at a specific Ub chain (indicated by a chain of coloured circles), this means the E3 ligase/DUB is known to regulate the protein by targeting this specific chain; E3 ligases always add Ub chains while DUBs always remove Ub chains. Arrows always indicate positive regulation of a protein they point at (or the protein at the end of the Ub chain they point at), while flat-headed lines indicate the opposite; however, due to FLIP(L)’s controversial signalling, lines pointing from E3 ligases/DUBs to FLIP indicate whether they induce stabilisation (arrows) or degradation (flat-heads) of FLIP.

The complexes formed following ligation of TNFR1 are well characterised, and the role of ubiquitination studied in depth. It is thought that stimulated TNFR1 recruits RIPK1 and TRADD, then TRADD recruits TRAF2/5, which in turn recruits cIAP1/2 [65]. As E3 ligases, cIAP1/2 can ubiquitinate multiple components of Complex I with K63-linked chains, including RIPK1; subsequently, the E3 ligase linear ubiquitination chain assembly complex (LUBAC) is recruited to the complex and adds M1-linked chains to RIPK1 [20, 66–68]. Both types of chains present on RIPK1 lead to recruitment of the TAK1 and IKK complexes, and subsequent activation of the MAPK and NF-κB pathways, respectively. Additionally, cIAP1 was shown to modify RIPK1 with K11-linked chains, enhancing recruitment of the IKK complex [69]. This complex of proteins described so far is named Complex I; however, if RIPK1 is either deubiquitinated by CYLD [70] or not ubiquitinated when recruited to Complex I (for example, in the context of IAP depletion [65]) then Complex IIa or IIb is formed, respectively. Depending on the cellular conditions, both Complex IIa/b can induce apoptosis or necroptosis [71] (Fig. 3).

#### TRAIL-R-induced complexes

**Fig. 4:**
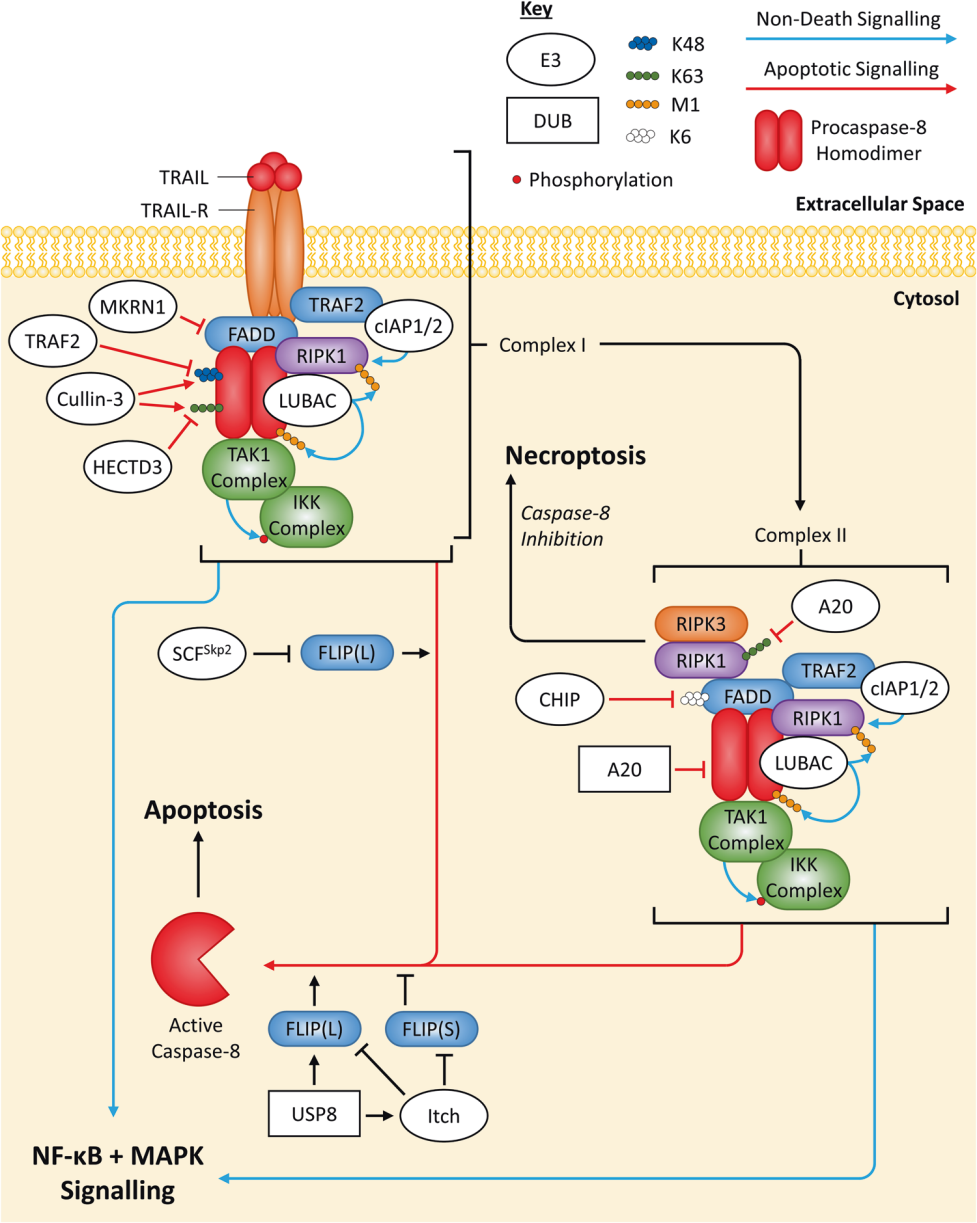
Overview of ubiquitin-mediated regulation of TRAIL-R apoptotic signalling. When trimeric TRAIL binds to trimeric TRAIL-R, Complex I is formed through protein:protein interactions and possibly through interactions mediated through Ub chains. Additionally, Complex I is thought to be able to dissociate from TRAIL-R and form Complex II in the cytosol; this has a similar composition to Complex I. Both Complex I and II can activate NF-κB, MAPK and apoptotic signalling, while only Complex II can also activate necroptosis. The exact recruitment process of proteins within Complex I/II is not known; however, Caspase-8’s scaffolding function seems to be important for the interaction of FADD, FLIP(L/S), RIPK1, LUBAC, the TAK1 complex and the IKK complex within Complex I/II, while TRAF2 and cIAP1/2 appear to associate with TRAIL-R. It is not known how TRAF2 and cIAP1/2 associate with Complex II so, for convenience, they have been shown to interact with FADD. E3 ligases/DUBs that regulate Complex I/II do not necessarily regulate each complex specifically, it is for convenience they have been drawn this way. Regulation by E3 ligases (white ovals) and deubiquitinating enzymes DUBs (white rectangles) are indicated. If an E3 ligase/DUB is pointing at a specific Ub chain (indicated by a chain of coloured circles), this means the E3 ligase/DUB is known to regulate the protein by targeting this specific chain; E3 ligases always add Ub chains while DUBs always remove Ub chains. Arrows always indicate positive regulation of a protein they point at (or the protein at the end of the Ub chain they point at), while flat-headed lines indicate the opposite; however, due to FLIP(L)’s controversial signalling, lines pointing from E3 ligases/DUBs to FLIP indicate whether they induce stabilisation (arrows) or degradation (flat-heads) of FLIP. Line colour also indicates the type of signalling that is regulated, either apoptotic (red) or non-death (blue) signalling.

More recently, TRAIL-R simulation has been shown to induce formation of complexes containing similar proteins to TNFR1-induced complexes, with analogous regulation via ubiquitination. In TRAIL-R signalling, it is thought that pro-apoptotic proteins (FADD and Caspase-8) are recruited to TRAIL-R first and can act as a scaffold for anti-apoptotic protein recruitment (RIPK1, TRAF2, cIAP1/2, LUBAC, the TAK1 and IKK complexes) [1, 72, 73]. The complex described so far is termed Complex I and is thought to be able to dissociate from the activated TRAIL-R and form a complex in the cytosol, named Complex II, which has a very similar composition to Complex I [74]. Both TRAIL-R-induced Complex I and II can activate NF-κB, MAPK and apoptotic pathways, but only Complex II can activate necroptosis as well [1] (Fig. 4). In this review, the death-inducing signalling complex (DISC) will be used to refer the complex of proteins within TRAIL-induced Complex I/II that specifically induces apoptosis. The exact spatial and temporal location of the proteins during the TRAIL-R signalling cascade is not well defined, compared to TNFR1 signalling. Additionally, it is unclear what determines whether Complex I/II induces non-apoptotic vs apoptotic signalling; however, M1-linked ubiquitination of Caspase-8 by LUBAC has been shown to suppress its activity [75], potentially allowing propagation of pro-survival and inflammatory pathways that are normally inhibited by Caspase-8 activity [73, 76].

#### The DED proteins: FADD

Death effect domain (DED)-containing proteins are the mediators of death receptor-induced apoptosis in complexes such as the DISCs of the TRAIL-R-induced Complex I and II, and the TNFR1-induced Complex IIa/b. The two DED proteins essential to this process are FADD and Caspase-8, FADD being an adaptor protein that mediates the recruitment of Procaspase-8 and allowing Procaspase-8 to homodimerize and fully activate. Active Caspase-8 is cytosolically free and able to cleave Procaspase-3/7, initiating apoptosis.

Recently FADD has been shown to be ubiquitinated by the E3 ligase CHIP [77]. Using mass spectrometry, FADD’s K149 and K153 residues, located in its C-terminal death domain (DD), were identified as ubiquitination sites and, when mutated simultaneously, inhibited CHIP-mediated ubiquitination of FADD in vitro (cell free) [77]. Additionally, implementing in vitro assays (with FADD, CHIP and mutated versions of Ub), FADD was shown to only be modified with K6-linked chains via CHIP and double mutation of K149/153 increased cell’s sensitivity to TRAIL-induced apoptosis [77]. Interestingly, double mutation of K149/153 did not affect FADD’s interaction with other DD-containing proteins, like TRAIL-R2, but did exert more potent apoptotic effects, which was mimicked by loss of CHIP [77].

FADD has also been shown to undergo M1-linked ubiquitination, dependent on the catalytic subunit of LUBAC, named HOIP [78]. Jurkat cells were shown to induce FADD M1-linked ubiquitination when treated with TNF-α alone; however, during TNF-α-induced apoptosis, initial M1-linked ubiquitination gradually decreased overtime, similar to NEMO (a known substrate of LUBAC and part of the IKK complex) [78]. It was also shown that HOIP is cleaved by Caspase-8 in apoptotic cells, indicating that M1-linked ubiquitination of FADD represses pro-apoptotic signalling of TNFR1 [78].

#### The DED proteins: FLIP

Caspase-8’s activation is known to be regulated by its paralog, FLIP, which is expressed as two splice variants, FLIP Long (FLIP(L)) and FLIP Short (FLIP(S)). Any complexes mentioned so far that include FADD and Caspase-8 are usually accompanied by FLIP(L/S). FLIP(L/S) can form heterodimers with Procaspase-8 and were traditionally thought to both inhibit Caspase-8 activation. However, while the FLIP(S):Procaspase-8 heterodimer has no catalytic activity and potently inhibits Caspase-8 activation, the FLIP(L):Procaspase-8 heterodimer does have catalytic activity (that is spatially restricted [79]) and is able to promote Caspase-8 activation, depending on its relative levels to Procaspase-8 [79–85]. Overexpression of FLIP(L) will block fully active Caspase-8 from forming and inhibit apoptosis. Like Mcl-1 and Bim, FLIP expression is tightly regulated by the UPS [86–88].

Mass spectrometry identified K351 and K353 as potential ubiquitination sites within FLIP(L), specifically when LUBAC was overexpressed [89]. Interestingly, during TNF-α-induced apoptosis FLIP(L)^K351R/K353R^ was detected with fewer M1-linked chains but had an increase in K48-linked chains (the opposite to wild-type FLIP(L)); along with data showing decreased FLIP(L) stability in HOIP knock out cells, this study concluded that M1-linked chains are able to inhibit degradative K48-linked chains from being conjugated to FLIP(L), leading to FLIP(L) stabilisation during TNF-α-induced apoptosis [89].

Recently, the SCF^Skp2^ E3 ligase complex was shown to interact with FLIP(L) and induce its proteasomal degradation, while a stronger interaction with the Caspase-8 processed form of FLIP(L), p43-FLIP(L), could also be detected [82]. p43-FLIP(L) is predominantly localised at the DISC and it was therefore not surprising that the SCF^Skp2^ complex was also found to associate with TRAIL-R2 and dampen TRAIL-induced apoptosis [82]. Furthermore, competition for p43-FLIP(L) between the SCF^Skp2^ complex and DISC components (FADD and Procaspase-8) was demonstrated, overall suggesting that the SCF^Skp2^ complex competes with proteins within the DISC for an interaction with p43-FLIP(L) and induces its ubiquitination-dependent proteasomal degradation [82].

The E3 ligase MIB2 was recently shown to stabilise FLIP(L), resulting in reduced TNF-α-induced apoptosis [90]. In vitro analysis combined with antibodies specific for K48- and K63-linked chains showed that MIB2 was able to conjugate both these chain types onto FLIP(L) [90]. K48-linked chains are usually associated with degradation of a target protein; however, the possibility of branched chains might explain the effect of MIB2 on FLIP(L) [22]. Since FLIP(L) can promote Caspase-8 activation [79–85], the study hypothesised that MIB2 ubiquitination of FLIP(L)’s Caspase-like domain could be interfering with the FLIP(L):Procaspase-8 interaction and, therefore, processing of Procaspase-8 [90].

### Necroptosis

Necroptosis is a form of programmed necrosis that is Caspase-independent, more pro-inflammatory than apoptosis [91, 92] and is characterised by necrotic morphological changes, including cellular/organelle swelling and rupturing of the plasma membrane [92]. The most well-characterised pathway to induce necroptosis is by TNFR1 signalling via Complex IIb and will be the main pathway focused on in this review. As TNF-α stimulation of TNFR1 alone does not form Complex IIb, cells are frequently co-treated with IAP inhibitors (also known as SMAC mimetics) in combination with TNF-α [65]. Caspase-8 activity from within Complex IIb, often driven primarily by the Caspase-8:FLIP(L) heterodimer, cleaves RIPK1 and RIPK3 within Complex IIb and is thought to inhibit necrosome formation; however, the Caspase-8:FLIP(S) heterodimer has no enzymatic activity and is unable to cleave RIPK1/3, thereby promoting necroptosis [93, 94]. A lot of studies use the pan-caspase inhibitor zVAD-fmk to inhibit Caspase-8 activity and combine it with TNF-α and SMAC mimetics to induce necroptosis.

#### Necrosome formation: RIPK1 and RIPK3 dimerisation

**Fig. 5:**
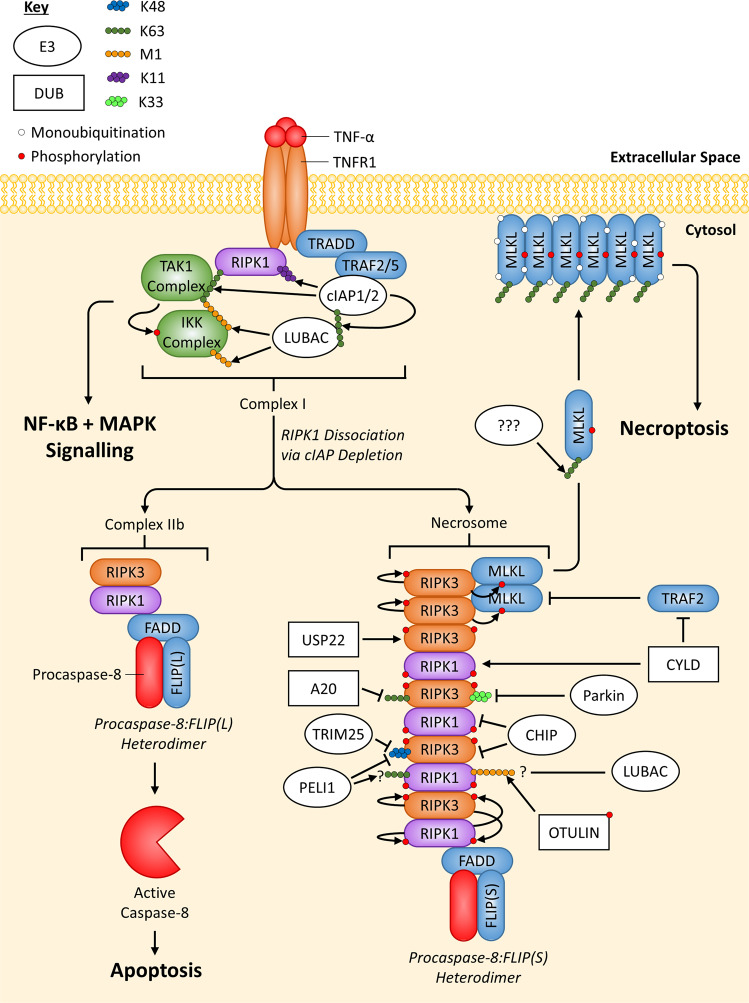
Overview of ubiquitin-mediated regulation of TNFR1 necroptotic signalling. When non-ubiquitinated RIPK1 dissociates from TNFR1-induced Complex I, it can form TNFR1-induced Complex IIb. Caspase-8 activity, present in the Procaspase-8:FLIP(L) heterodimer, can negatively regulate RIPK1/3 and inhibit necroptosis, while also regulating apoptosis. However, a lack of Caspase-8 activity within Complex IIb, either from the Procaspase-8:FLIP(S) heterodimer, expression of viral FLIP or treatment with a caspase inhibitor (e.g. zVAD-fmk), leads to phosphorylation and oligomerisation of RIPK1:RIPK3 heterodimers, which can induce the recruitment and phosphorylation of RIPK3 homodimers. RIPK3 homodimers can then recruit and phosphorylate MLKL, which then translocates/oligomerises to the plasma membrane, inducing necroptosis. Complex IIb ubiquitination events have not been highlighted since they are covered in Fig. 3. Regulation by E3 ligases (white ovals) and deubiquitinating enzymes DUBs (white rectangles) are indicated. If an E3 ligase/DUB is pointing at a specific Ub chain (indicated by a chain of coloured circles), this means the E3 ligase/DUB is known to regulate the protein by targeting this specific chain; E3 ligases always add Ub chains while DUBs always remove Ub chains. Arrows always indicate positive regulation of a protein they point at (or the protein at the end of the Ub chain they point at), while flat-headed lines indicate the opposite.

RIPK1 and RIPK3 are able to heterodimerise via their RIP homotypic interaction motif (RHIMs) and are found within Complex IIb [95, 96]. When complexed with one another, RIPK1 and RIPK3 auto-phosphorylate and phosphorylate one other, which is essential for their interaction and induction of necroptosis [95, 97]. If Caspase-8 activity is inhibited, the amyloid structured necrosome can form, composed predominantly of RIPK1 and RIPK3 heterodimers [96]. Within the necrosome, RIPK3 can recruit cytosolically free RIPK3 and form a homodimer that induces RIPK3 autophosphorylation, necessary for RIKP3 to recruit and phosphorylate MLKL [98, 99] (Fig. 5).

While RIPK1 phosphorylation is well known to be crucial for necroptosis, in the context of TNF-α signalling [97], ubiquitination of RIPK1 has also been demonstrated to occur in a necroptosis-specific manner. Using mass spectrometry to identify RIPK1 ubiquitination sites in HT29 cells, K115 of RIPK1 was found to be specifically ubiquitinated during TNF-α-induced necroptosis [100]. To date, M1-/K63-linked Ub chains seem to be predominantly conjugated to RIPK1 during TNF-α-induced necroptosis and RIPK1’s K115 residue may be the sole site for these conjugates specifically during necroptosis [100–103]. However, it is unclear how these two chain types simultaneously occur at one site and, while mutation of K115 dampens necroptosis, if both M1- and K63-linked chains conjugated to K115 are both pro-necroptotic or if one chain type is pro- and the other is anti-necroptotic.

The M1-linked ubiquitination of RIPK1 within the necrosome/Complex IIb was also shown to be reliant on LUBAC; however, LUBAC did not seem to affect necrosome formation or its signalling output [101]. Recently, the DUB OTULIN was shown to remove M1-linked chains from RIPK1 during necroptosis and, while not affecting necrosome formation, increased TNF-α-induced necroptosis [103]. Additionally, OTULIN was shown to be phosphorylated at Try56 during TNF-α-induced necroptosis and mutation of this site abrogated OTULIN-dependent M1-linked deubiquitination of RIPK1 and supressed TNF-α-induced necroptosis [103]. Overall, this indicates that M1-linked ubiquitination of RIPK1 could repress necroptosis [103].

Mutation of K612 of mouse RIPK1 disrupted its DD-mediated interactions with other DD-containing proteins, such as FADD and TNFR1 [104]. K612 mutation reduced RIPK1 recruitment to TNFR1-induced Complex I in addition to reducing RIPK1 activation and disrupting the overall ubiquitination pattern of RIPK1, possibly due to the alteration of Complex I [104]. The consequence of this mutation was reduced NF-κB and MAPK signalling, in addition to inhibition of apoptosis and necroptosis due to TNFR1-induced Complex II not being able to form from a disrupted Complex I [104].

Studies have also suggested that RIPK3 ubiquitination is involved in necroptosis propagation. The DUB activity of A20 was shown to inhibit RIPK3 ubiquitination and reduce the RIPK1:RIPK3 interaction, thereby suppressing TNF-α-induced necroptosis [105]. Mass spectrometry identified K5 of RIPK3 as a ubiquitination site, specifically in *A20*^*-/-*^ mouse cells undergoing necroptosis, and, using cell lines stably expressing RIPK3^K5A^, was also shown to slightly increase RIPK1:RIPK3 complexes during TNF-α-induced necroptosis [105]. RIPK3 was shown to undergo K63-linked ubiquitination in *A20*^*-/-*^ mouse cells and vice versa in HEK293T cells overexpressing RIPK3 and A20; however, this was not demonstrated specifically for RIPK3’s K5 residue [105].

Necroptosis-induced K518 ubiquitination of RIPK3 reduces its interaction with the necrosome and dampens necroptosis propagation, which was shown to be reversed by the DUB USP22 [106]. Unusually, the inhibitory ubiquitin chains conjugated to K518 of RIPK3 increase RIPK3 phosphorylation, which is counter-intuitive since phosphorylated RIPK3 is indicative of its activation at the necrosome [106].

The E3 ligase Parkin has also been implicated in RIPK3 ubiquitination and regulation of necroptosis driven by TNF-α [107]. Under necroptotic stimuli, AMPK phosphorylates Ser9 of Parkin, activating Parkin’s E3 ligase activity [107]. RIPK3’s K197, K302 and K364 residues were shown to be ubiquitination sites for Parkin; additionally, simultaneous mutation of these residues substantially decreased its overall ubiquitination [107]. K33-linked chains were the only chains found to be conjugated to RIPK3 in a Parkin-dependent manner; however, overexpression of mutant Ub was used for all of these experiments, so some caution should be taken when interpreting this result [107].

The E3 ligase CHIP, known to suppress TRAIL-induced apoptosis by ubiquitinating FADD [77], was shown to ubiquitinate both RIPK1 and RIPK3, inducing their degradation via lysosomes [108]. K55 and K363 of RIPK3 were identified as the ubiquitination sites for CHIP-mediated ubiquitination, and knock out of CHIP in mouse cells was shown to increase TNF-α-induced necroptosis [108]. The stabilisation of RIPK3 was suggested to be the factor causing increased necroptosis sensitivity induced by loss of CHIP [108]; however, both RIPK1 and RIPK3 were stabilised with CHIP knock out [108] and the ratio, rather than the total levels, of RIPK1 and RIPK3 seems to determine sensitivity of cells to necroptosis [99]. Nevertheless, CHIP does appear to protect cells from TNF-α-induced necroptosis, possibly by reducing the levels of both RIPK1 and RIPK3 [108].

Another E3 ligase, TRIM25, was shown to induce degradation of RIPK3 via K48-linked chains [109]. Mutation of K501 of RIPK3 markedly reduced its TRIM25-dependent ubiquitination and increased TNF-α-induced necroptosis, indicating that TRIM25 is anti-necroptotic by reducing the expression of RIPK3 via proteasomal degradation [109]. However, overexpression experiments of mutant Ub were used to determine chain types conjugated to RIPK3, so should be interpreted with caution.

Additionally, the E3 ligase PELI1 was shown to interact with and ubiquitinate K363 of RIPK3 (like CHIP) with K48-linked chains, inducing proteasomal degradation of RIPK3; however, chain type was determined with overexpression of mutant Ub and should, therefore, be cautiously interpreted [110]. T182 was identified as a potential autophosphorylation site within RIPK3, the phosphorylation of which was necessary for autophosphorylation of S227 within RIPK3, a well-known marker of active RIPK3 [110]. Unlike CHIP, PELI1 only interacts with and degrades RIPK3 when T182 is phosphorylated and PELI1 is found in complex with RIPK1/3 during necroptosis, altogether indicating that PELI1 interacts only with active RIPK3 [110]. Considering that PELI1 can stop spontaneous necroptosis induced by high levels of RIPK3, it is thought that, in addition to suppressing TNF-α-induced necroptosis, PELI1 might act as a safeguard that targets spontaneously autophosphorylated RIPK3 for proteasomal degradation under basal conditions [110]. However, to complicate matters, another study using PELI1 knock out cells reported that PELI1 promoted necroptosis and concluded that it was due to it acting as an E3 ligase for RIPK1 [102]. While these conflicting results cannot explain why two separate studies using similar PELI1 knock out cells concluded opposite effects on necroptotic propagation, only the RIPK3-focussed study demonstrated direct ubiquitination of RIPK3 by PELI1 via an in vitro ubiquitination assay [110]. It is possible that the interaction of RIPK1 with PELI1 is indirect and mediated via RIPK3; indeed, mapping studies indicated that the RHIM domain of RIPK1 is critical for its interaction with PELI1; this domain being crucial for the RIPK1:RIPK3 interaction [102].

#### MLKL recruitment, translocation and oligomerisation

Homodimerised RIPK3 within the necrosome is able to recruit and phosphorylate MLKL, necessary for MLKL activation [98, 99]. In mouse models, ubiquitinated TRAF2 was shown to associate with inactive MLKL and inhibit MLKL’s interaction with other components of the necrosome, independent of TRAF2’s E3 ligase activity [111]. However, TNF-α-induced necroptosis reduces TRAF2 ubiquitination and suppresses the TRAF2:MLKL interaction; therefore, allowing MLKL to propagate necroptosis [111]. In past studies, the DUB CYLD was reported to promote necroptosis via deubiquitination of RIPK1 [112, 113]; but recently, CYLD was also shown to be responsible for deubiquitinating TRAF2 during necroptosis, stopping TRAF2’s inhibition of MLKL [111]. Considering that CYLD is negatively regulated by Caspase-8-mediated processing (like other necroptosis promoting proteins) [112], this is a further indication of its importance in necroptotic propagation.

After MLKL has been recruited to the necrosome and phosphorylated by RIPK3, this results in the oligomerisation and translocation of MLKL to the plasma membrane, where it is able to form higher-order structures [114, 115]. It is not completely clear how MLKL induces necrosis, but there are currently two non-exclusive models: either, MLKL acts as a platform at the plasma membrane to recruit Ca^2+^ or Na^+^ ion channels [116, 117], or MLKL forms part of a complex that creates a pore in the membrane [114, 118].

Mass spectrometry identified K219 as a ubiquitination site of mouse MLKL and mutation of the site was shown to reduce K63-linked ubiquitination of MLKL, generated during TNF-α-induced necroptosis [119]. Additionally, ubiquitinated K219 was shown to predominantly promote necroptosis in mouse dermal fibroblasts (MDFs) derived from *Mlkl*^*K219R/K219R*^ knock-in mice, which almost completely blocked TNF-α-induced necroptosis, and abrogated phenotypes associated with necroptosis-induced tissue injury were seen in *Mlkl*^*K219R/K219R*^ mice [119]. Interestingly, *Mlkl*^*K219R/K219R*^ MDFs cannot form nearly as many higher-order MLKL polymers in membrane fractions compared to wild-type MDFs, indicating that K219 ubiquitination promotes MLKL oligomerisation at the plasma membrane [119]. In contrast, another study showed that exogenous expression of a non-ubiquitinated MLKL-USP21 fusion protein (USP21 removes all Ub conjugates from a protein) had minimal effects on TNF-α-induced necroptosis, but did affect MLKL stability [120]. Additionally, this study also concluded that MLKL was multi-monoubiquitinated (via UbiCRest) when recruited to the membrane during necroptosis [120], but the effects of this type of ubiquitination on necroptosis was unclear.

A summary of the ubiquitination events reported to occur during necroptosis that are covered in this review can be found in Table 1.

**Table 1:** Necroptotic proteins and the ubiquitination machinery that regulates them during TNF-α-induced necroptosis.

Necroptotic protein	Conjugation site (Ms = Mouse)	E3 Ligase*/ DUB** (and Chain type targeted)	Impact on death receptor signalling	References
RIPK1	K115	Pellino-1???* (K63)	↑Necroptosis?	[100, 102]
		???* (M1)	↑Necroptosis?	[100]
	K376 (Ms)	cIAP1/2* (K63)	↑Non-death, ↓Apoptosis, ↓Necroptosis	[125, 126]
	K612 (Ms)	???*	↑Non-death, ↑Apoptosis, ↑Necroptosis	[104]
	???	LUBAC* (M1)	No effect?	[101]
		OTULIN** (M1)	↑Necroptosis	[103]
		CHIP*	↓Necroptosis	[108]
		CYLD**	↑Necroptosis	[112, 113]
RIPK3	K5 (Ms)	A20** (K63)	↓Necroptosis	[105]
	K55, K363	CHIP*	↓Necroptosis	[108]
	K363	Pellino-1* (K48)	↓Necroptosis	[110]
	K197, K302, K364	Parkin* (K33)	↓Necroptosis	[107]
	K501	TRIM25*(K48)	↓Necroptosis	[109]
	K518	USP22**	↑Necroptosis	[106]
MLKL	K219 (Ms)	???* (K63)	↑Necroptosis	[119]
	???	???*(Multi-monoubiquitination)	↓Necroptosis?	[120]
TRAF2	???	CYLD**	↑Necroptosis	[111]

*E3 Ligase, **DUB.

### Concluding remark

It is evident that ubiquitination plays an important role in regulating both the apoptotic and necroptotic pathways; elucidation of the mechanisms underlying ubiquitin-mediated control of cell death could provide evidence for the development of novel treatments for diseases that have dysregulated cell death pathways such as cancer [121] and neurological diseases [122, 123]. One unanswered question is the mechanism of how non-degradative Ub chains impact Death Receptor-induced complexes’ formation, such as TRAIL-induced Complex I/II and the necrosome. Hopefully development of new tools to detect branched Ub chains will alleviate the gap in the knowledge around this area.

## Supplementary information

Reproducibility Checklist form

## References

[1] Lafont E, Hartwig T, Walczak H (2017). Paving TRAIL’s path with ubiquitin. Trends Biochem Sci.

[2] Schlesinger DH, Goldstein G, Niall HD (1975). The complete amino acid sequence of ubiquitin, an adenylate cyclase stimulating polypeptide probably universal in living cells. Biochemistry.

[3] Hershko A (1996). Lessons from the discovery of the ubiquitin system. Trends Biochem Sci.

[4] Ye Y, Rape M (2009). Building ubiquitin chains: E2 enzymes at work. Nat Rev Mol Cell Biol.

[5] Duda DM, Olszewski JL, Schuermann JP, Kurinov I, Miller DJ, Nourse A (2013). Structure of HHARI, a RING-IBR-RING ubiquitin ligase: autoinhibition of an ariadne-family E3 and insights into ligation mechanism. Structure.

[6] Riley BE, Lougheed JC, Callaway K, Velasquez M, Brecht E, Nguyen L (2013). Structure and function of Parkin E3 ubiquitin ligase reveals aspects of RING and HECT ligases. Nat Commun.

[7] Stieglitz B, Rana RR, Koliopoulos MG, Morris-Davies AC, Schaeffer V, Christodoulou E (2013). Structural basis for ligase-specific conjugation of linear ubiquitin chains by HOIP. Nature.

[8] Mabbitt PD, Loreto A, Déry M-A, Fletcher AJ, Stanley M, Pao K-C (2020). Structural basis for RING-Cys-Relay E3 ligase activity and its role in axon integrity. Nat Chem Biol.

[9] Ahel J, Fletcher A, Grabarczyk DB, Roitinger E, Deszcz L, Lehner A, et al. E3 ubiquitin ligase RNF213 employs a non-canonical zinc finger active site and is allosterically regulated by ATP. bioRxiv. 2021. 10.1101/2021.05.10.443411.

[10] Komander D, Clague MJ, Urbé S (2009). Breaking the chains: structure and function of the deubiquitinases. Nat Rev Mol Cell Biol.

[11] Ventii KH, Wilkinson KD (2008). Protein partners of deubiquitinating enzymes. Biochemical J.

[12] Jura N, Scotto-Lavino E, Sobczyk A, Bar-Sagi D (2006). Differential modification of Ras proteins by ubiquitination. Mol Cell.

[13] Fallon L, Bélanger CML, Corera AT, Kontogiannea M, Regan-Klapisz E, Moreau F (2006). A regulated interaction with the UIM protein Eps15 implicates parkin in EGF receptor trafficking and PI(3)K-Akt signalling. Nat Cell Biol.

[14] Su Y-T, Gao C, Liu Y, Guo S, Wang A, Wang B (2013). Monoubiquitination of Filamin B regulates vascular endothelial growth factor-mediated trafficking of histone deacetylase 7. Mol Cell Biol.

[15] Wang X, Jin C, Tang Y, Tang LY, Zhang YE (2013). Ubiquitination of tumor necrosis factor receptor-associated factor 4 (TRAF4) by smad ubiquitination regulatory factor 1 (Smurf1) regulates motility of breast epithelial and cancer cells. J Biol Chem.

[16] Bienko M, Green CM, Sabbioneda S, Crosetto N, Matic I, Hibbert RG (2010). Regulation of translesion synthesis DNA polymerase η by monoubiquitination. Mol Cell.

[17] Datta AB, Hura GL, Wolberger C (2009). The structure and conformation of Lys63-Linked tetraubiquitin. J Mol Biol.

[18] Komander D, Reyes-Turcu F, Licchesi JDF, Odenwaelder P, Wilkinson KD, Barford D (2009). Molecular discrimination of structurally equivalent Lys 63‐linked and linear polyubiquitin chains. EMBO Rep.

[19] Eddins MJ, Varadan R, Fushman D, Pickart CM, Wolberger C (2007). Crystal structure and solution NMR studies of Lys48-linked tetraubiquitin at neutral pH. J Mol Biol.

[20] Emmerich CH, Bakshi S, Kelsall IR, Ortiz-Guerrero J, Shpiro N, Cohen P (2016). Lys63/Met1-hybrid ubiquitin chains are commonly formed during the activation of innate immune signalling. Biochem Biophys Res Commun.

[21] Emmerich CH, Ordureau A, Strickson S, Arthur JSC, Pedrioli PGA, Komander D (2013). Activation of the canonical IKK complex by K63/M1-linked hybrid ubiquitin chains. Proc Natl Acad Sci USA.

[22] Ohtake F, Tsuchiya H, Saeki Y, Tanaka K (2018). K63 ubiquitylation triggers proteasomal degradation by seeding branched ubiquitin chains. Proc Natl Acad Sci USA.

[23] Benard G, Neutzner A, Peng G, Wang C, Livak F, Youle RJ (2010). IBRDC2, an IBR-type E3 ubiquitin ligase, is a regulatory factor for Bax and apoptosis activation. EMBO J.

[24] Chen HC, Kanai M, Inoue-Yamauchi A, Tu HC, Huang Y, Ren D (2015). An interconnected hierarchical model of cell death regulation by the BCL-2 family. Nat Cell Biol.

[25] Ramesh P, Medema JP (2020). BCL-2 family deregulation in colorectal cancer: potential for BH3 mimetics in therapy. Apoptosis.

[26] Akiyama T, Bouillet P, Miyazaki T, Kadono Y, Chikuda H, Chung UI (2003). Regulation of osteoclast apoptosis by ubiquitylation of proapoptotic BH3-only Bcl-2 family member Bim. EMBO J.

[27] Wiggins CM, Band H, Cook SJ (2007). c-Cbl is not required for ERK1/2-dependent degradation of BimEL. Cell Signal.

[28] Zhang W, Cheng GZ, Gong J, Hermanto U, Zong CS, Chan J (2008). RACK1 and CIS mediate the degradation of BimEL in cancer cells. J Biol Chem.

[29] Thompson S, Pearson AN, Ashley MD, Jessick V, Murphy BM, Gafken P (2011). Identification of a novel Bcl-2-interacting mediator of cell death (Bim) E3 ligase, tripartite motif-containing protein 2 (TRIM2), and its role in rapid ischemic tolerance-induced neuroprotection. J Biol Chem.

[30] Dehan E, Bassermann F, Guardavaccaro D, Vasiliver-Shamis G, Cohen M, Lowes KN (2009). βTrCP- and Rsk1/2-mediated degradation of BimEL inhibits apoptosis. Mol Cell.

[31] Moustafa-Kamal M, Gamache I, Lu Y, Li S, Teodoro JG (2013). BimEL is phosphorylated at mitosis by Aurora A and targeted for degradation by βtrCP1. Cell Death Differ.

[32] Weber A, Heinlein M, Dengjel J, Alber C, Singh PK, Häcker G (2016). The deubiquitinase Usp27x stabilizes the BH 3‐only protein Bim and enhances apoptosis. EMBO Rep.

[33] Llambi F, Moldoveanu T, Tait SWG, Bouchier-Hayes L, Temirov J, McCormick LL (2011). A unified model of mammalian BCL-2 protein family interactions at the mitochondria. Mol Cell.

[34] Schwickart M, Huang X, Lill JR, Liu J, Ferrando R, French DM (2010). Deubiquitinase USP9X stabilizes MCL1 and promotes tumour cell survival. Nature.

[35] Zhang S, Zhang M, Jing Y, Yin X, Ma P, Zhang Z, et al. Deubiquitinase USP13 dictates MCL1 stability and sensitivity to BH3 mimetic inhibitors. Nat Commun. 2018;9. 10.1038/s41467-017-02693-9. 10.1038/s41467-017-02693-9 PMC5768685 29335437

[36] Magiera MM, Mora S, Mojsa B, Robbins I, Lassot I, Desagher S (2013). Trim17-mediated ubiquitination and degradation of Mcl-1 initiate apoptosis in neurons. Cell Death Differ.

[37] Zhong Q, Gao W, Du F, Wang X (2005). Mule/ARF-BP1, a BH3-only E3 ubiquitin ligase, catalyzes the polyubiquitination of Mcl-1 and regulates apoptosis. Cell.

[38] Inoue S, Hao Z, Elia AJ, Cescon D, Zhou L, Silvester J (2013). Mule/Huwe1/Arf-BP1 suppresses Ras-driven tumorigenesis by preventing c-Myc/Miz1-mediated down-regulation of p21 and p15. Genes Dev.

[39] Mei Y, Du W, Yang Y, Wu M (2005). Puma*Mcl-1 interaction is not sufficient to prevent rapid degradation of Mcl-1. Oncogene.

[40] Czabotar PE, Lee EF, Van Delft MF, Day CL, Smith BJ, Huang DCS (2007). Structural insights into the degradation of Mcl-1 induced by BH3 domains. Proc Natl Acad Sci USA.

[41] Warr MR, Mills JR, Nguyen M, Lemaire-Ewing S, Baardsnes J, Sun KLW (2011). Mitochondrion-dependent N-terminal processing of outer membrane Mcl-1 protein removes an essential Mule/Lasu1 protein-binding site. J Biol Chem.

[42] Gomez-Bougie P, Ménoret E, Juin P, Dousset C, Pellat-Deceunynck C, Amiot M (2011). Noxa controls Mule-dependent Mcl-1 ubiquitination through the regulation of the Mcl-1/USP9X interaction. Biochem Biophys Res Commun.

[43] Ren H, Koo J, Guan B, Yue P, Deng X, Chen M, et al. The E3 ubiquitin ligases β-TrCP and FBXW7 cooperatively mediates GSK3-dependent Mcl-1 degradation induced by the Akt inhibitor API-1, resulting in apoptosis. Mol Cancer. 2013;12. 10.1186/1476-4598-12-146. 10.1186/1476-4598-12-146 PMC3924345 24261825

[44] Harley ME, Allan LA, Sanderson HS, Clarke PR (2010). Phosphorylation of Mcl-1 by CDK1-cyclin B1 initiates its Cdc20-dependent destruction during mitotic arrest. EMBO J.

[45] Wu Y, Li X, Jia J, Zhang Y, Li J, Zhu Z (2018). Transmembrane E3 ligase RNF183 mediates ER stress-induced apoptosis by degrading Bcl-xL. Proc Natl Acad Sci USA.

[46] Rowland AA, Voeltz GK (2012). Endoplasmic reticulum-mitochondria contacts: Function of the junction. Nat Rev Mol Cell Biol.

[47] Metzger MB, Maurer MJ, Dancy BM, Michaelis S (2008). Degradation of a cytosolic protein requires endoplasmic reticulum-associated degradation machinery. J Biol Chem.

[48] Jost PJ, Grabow S, Gray D, McKenzie MD, Nachbur U, Huang DCS (2009). XIAP discriminates between type I and type II FAS-induced apoptosis. Nature.

[49] Ribeiro PS, Kuranaga E, Tenev T, Leulier F, Miura M, Meier P (2007). DIAP2 functions as a mechanism-based regulator of drICE that contributes to the caspase activity threshold in living cells. J Cell Biol.

[50] Silke J, Meier P. Inhibitor of apoptosis (IAP) proteins-modulators of cell death and inflammation. Cold Spring Harb Perspect Biol. 2013;5. 10.1101/cshperspect.a008730. 10.1101/cshperspect.a008730 PMC3552501 23378585

[51] Yang Y, Fang S, Jensen JP, Weissman AM, Ashwell JD (2000). Ubiquitin protein ligase activity of IAPs and their degradation in proteasomes in response to apoptotic stimuli. Science.

[52] Ryu YS, Lee Y, Lee KW, Hwang CY, Maeng JS, Kim JH (2011). TRIM32 protein sensitizes cells to tumor necrosis factor (TNFα)-induced apoptosis via its RING domain-dependent E3 ligase activity against X-linked Inhibitor of Apoptosis (XIAP). J Biol Chem.

[53] Zhou Z, Luo A, Shrivastava I, He M, Huang Y, Bahar I (2017). Regulation of XIAP turnover reveals a role for USP11 in promotion of tumorigenesis. EBioMedicine.

[54] Engel K, Rudelius M, Slawska J, Jacobs L, Ahangarian Abhari B, Altmann B (2016). USP9X stabilizes XIAP to regulate mitotic cell death and chemoresistance in aggressive B‐cell lymphoma. EMBO Mol Med.

[55] Edison N, Curtz Y, Paland N, Mamriev D, Chorubczyk N, Haviv-Reingewertz T (2017). Degradation of Bcl-2 by XIAP and ARTS promotes apoptosis. Cell Rep.

[56] Bornstein B, Gottfried Y, Edison N, Shekhtman A, Lev T, Glaser F (2011). ARTS binds to a distinct domain in XIAP-BIR3 and promotes apoptosis by a mechanism that is different from other IAP-antagonists. Apoptosis.

[57] Galbán S, Duckett CS (2010). XIAP as a ubiquitin ligase in cellular signaling. Cell Death Differ.

[58] Schile AJ, García-Fernández M, Steller H (2008). Regulation of apoptosis by XIAP ubiquitin-ligase activity. Genes Dev.

[59] Chai J, Du C, Wu JW, Kyin S, Wang X, Shi Y (2000). Structural and biochemical basis of apoptotic activation by Smac/DIABLO. Nature.

[60] Suzuki Y, Takahashi-Niki K, Akagi T, Hashikawa T, Takahashi R (2004). Mitochondrial protease Omi/HtrA2 enhances caspase activation through multiple pathways. Cell Death Differ.

[61] Garrison JB, Correa RG, Gerlic M, Yip KW, Krieg A, Tamble CM (2011). ARTS and Siah collaborate in a pathway for XIAP degradation. Mol Cell.

[62] Kim JB, Kim SY, Kim BM, Lee H, Kim I, Yun J (2013). Identification of a novel anti-apoptotic E3 ubiquitin ligase that ubiquitinates antagonists of inhibitor of apoptosis proteins SMAC, HtrA2, and ARTS. J Biol Chem.

[63] Falschlehner C, Emmerich CH, Gerlach B, Walczak H (2007). TRAIL signalling: decisions between life and death. Int J Biochem Cell Biol.

[64] Barnhart BC, Legembre P, Pietras E, Bubici C, Franzoso G, Peter ME (2004). CD95 ligand induces motility and invasiveness of apoptosis-resistant tumor cells. EMBO J.

[65] Bertrand MJM, Milutinovic S, Dickson KM, Ho WC, Boudreault A, Durkin J (2008). cIAP1 and cIAP2 facilitate cancer cell survival by functioning as E3 Ligases that promote RIP1 ubiquitination. Mol Cell.

[66] Gerlach B, Cordier SM, Schmukle AC, Emmerich CH, Rieser E, Haas TL (2011). Linear ubiquitination prevents inflammation and regulates immune signalling. Nature.

[67] Keusekotten K, Elliott PR, Glockner L, Fiil BK, Damgaard RB, Kulathu Y (2013). OTULIN antagonizes LUBAC signaling by specifically hydrolyzing met1-linked polyubiquitin. Cell.

[68] Fiil BK, Damgaard RB, Wagner SA, Keusekotten K, Fritsch M, Bekker-Jensen S (2013). OTULIN restricts Met1-linked ubiquitination to control innate immune signaling. Mol Cell.

[69] Dynek JN, Goncharov T, Dueber EC, Fedorova AV, Izrael-Tomasevic A, Phu L (2010). c-IAP1 and UbcH5 promote K11-linked polyubiquitination of RIP1 in TNF signalling. EMBO J.

[70] Kovalenko A, Chable-Bessia C, Cantarella G, Israël A, Wallach D, Courtois G (2003). The tumour suppressor CYLD negatively regulates NF-κB signalling by deubiquitination. Nature.

[71] Pasparakis M, Vandenabeele P (2015). Necroptosis and its role in inflammation. Nature.

[72] Dickens LS, Powley IR, Hughes MA, MacFarlane M (2012). The ‘complexities’ of life and death: death receptor signalling platforms. Exp Cell Res.

[73] Henry CM, Martin SJ (2017). Caspase-8 acts in a non-enzymatic role as a scaffold for assembly of a pro-inflammatory “FADDosome” complex upon TRAIL stimulation. Mol Cell.

[74] Varfolomeev E, Maecker H, Sharp D, Lawrence D, Renz M, Vucic D (2005). Molecular determinants of kinase pathway activation by Apo2 ligand/tumor necrosis factor-related apoptosis-inducing ligand. J Biol Chem.

[75] Lafont E, Kantari-Mimoun C, Draber P, De Miguel D, Hartwig T, Reichert M (2017). The linear ubiquitin chain assembly complex regulates TRAIL‐induced gene activation and cell death. EMBO J.

[76] Kreuz S, Siegmund D, Rumpf JJ, Samel D, Leverkus M, Janssen O (2004). NFκB activation by Fas is mediated through FADD, caspase-8, and RIP and is inhibited by FLIP. J Cell Biol.

[77] Seo J, Lee EW, Shin J, Seong D, Nam YW, Jeong M (2018). K6 linked polyubiquitylation of FADD by CHIP prevents death inducing signaling complex formation suppressing cell death. Oncogene.

[78] Goto E, Tokunaga F (2017). Decreased linear ubiquitination of NEMO and FADD on apoptosis with caspase-mediated cleavage of HOIP. Biochem Biophys Res Commun.

[79] Pop C, Oberst A, Drag M, Van Raam BJ, Riedl SJ, Green DR (2011). FLIP L induces caspase 8 activity in the absence of interdomain caspase 8 cleavage and alters substrate specificity. Biochem J.

[80] Boatright KM, Deis C, Denault J-B, Sutherlin DP, Salvesen GS (2004). Activation of caspases-8 and -10 by FLIP L. Biochem J.

[81] Humphreys LM, Fox JP, Higgins CA, Majkut J, Sessler T, McLaughlin K (2020). A revised model of TRAIL ‐R2 DISC assembly explains how FLIP (L) can inhibit or promote apoptosis. EMBO Rep.

[82] Roberts JZ, Holohan C, Sessler T, Fox J, Crawford N, Riley JS, et al. The SCFSkp2 ubiquitin ligase complex modulates TRAIL-R2-induced apoptosis by regulating FLIP(L). Cell Death Differ. 2020;27:2726–41. 10.1038/s41418-020-0539-7 PMC7429845 32313199

[83] Fricker N, Beaudouin J, Richter P, Eils R, Krammer PH, Lavrik IN (2010). Model-based dissection of CD95 signaling dynamics reveals both a pro- and antiapoptotic role of c-FLIPL. J Cell Biol.

[84] Hughes MA, Powley IR, Jukes-Jones R, Horn S, Feoktistova M, Fairall L (2016). Co-operative and hierarchical binding of c-FLIP and Caspase-8: a unified model defines how c-FLIP isoforms differentially control cell fate. Mol Cell.

[85] Yu JW, Jeffrey PD, Shi Y (2009). Mechanism of procaspase-8 activation by c-FLIPL. Proc Natl Acad Sci USA.

[86] Poukkula M, Kaunisto A, Hietakangas V, Denessiouk K, Katajamäki T, Johnson MS (2005). Rapid turnover of c-FLIPshort is determined by its unique C-terminal tail. J Biol Chem.

[87] Kaunisto A, Kochin V, Asaoka T, Mikhailov A, Poukkula M, Meinander A (2009). PKC-mediated phosphorylation regulates c-FLIP ubiquitylation and stability. Cell Death Differ.

[88] Wilkie-Grantham RP, Matsuzawa S-II, Reed JC (2013). Novel phosphorylation and ubiquitination sites regulate reactive oxygen species-dependent degradation of anti-apoptotic c-FLIP protein. J Biol Chem.

[89] Tang Y, Joo D, Liu G, Tu H, You J, Jin J (2018). Linear ubiquitination of cFLIP induced by LUBAC contributes to TNF-induced apoptosis. J Biol Chem.

[90] Nakabayashi O, Takahashi H, Moriwaki K, Komazawa-Sakon S, Ohtake F, Murai S (2021). MIND bomb 2 prevents RIPK1 kinase activity-dependent and -independent apoptosis through ubiquitylation of cFLIPL. Commun Biol.

[91] Kaczmarek A, Vandenabeele P, Krysko DV (2013). Necroptosis: the release of damage-associated molecular patterns and its physiological relevance. Immunity.

[92] Liu C, Zhang K, Shen H, Yao X, Sun Q, Chen G (2018). Necroptosis: a novel manner of cell death, associated with stroke (review). Int J Mol Med.

[93] Tenev T, Bianchi K, Darding M, Broemer M, Langlais C, Wallberg F (2011). The ripoptosome, a signaling platform that assembles in response to genotoxic stress and loss of IAPs. Mol Cell.

[94] Feoktistova M, Geserick P, Kellert B, Dimitrova DP, Langlais C, Hupe M (2011). CIAPs block ripoptosome formation, a RIP1/Caspase-8 containing intracellular cell death complex differentially regulated by cFLIP isoforms. Mol Cell.

[95] Degterev A, Hitomi J, Germscheid M, Ch’en IL, Korkina O, Teng X (2008). Identification of RIP1 kinase as a specific cellular target of necrostatins. Nat Chem Biol.

[96] Li J, McQuade T, Siemer AB, Napetschnig J, Moriwaki K, Hsiao YS (2012). The RIP1/RIP3 necrosome forms a functional amyloid signaling complex required for programmed necrosis. Cell.

[97] Cho YS, Challa S, Moquin D, Genga R, Ray TD, Guildford M (2009). Phosphorylation-driven assembly of the RIP1-RIP3 complex regulates programmed necrosis and virus-induced inflammation. Cell.

[98] Orozco S, Yatim N, Werner MR, Tran H, Gunja SY, Tait SWG (2014). RIPK1 both positively and negatively regulates RIPK3 oligomerization and necroptosis. Cell Death Differ.

[99] Wu XN, Yang ZH, Wang XK, Zhang Y, Wan H, Song Y (2014). Distinct roles of RIP1-RIP3 hetero-and RIP3-RIP3 homo-interaction in mediating necroptosis. Cell Death Differ.

[100] De Almagro MC, Goncharov T, Izrael-Tomasevic A, Duttler S, Kist M, Varfolomeev E (2017). Coordinated ubiquitination and phosphorylation of RIP1 regulates necroptotic cell death. Cell Death Differ.

[101] De Almagro MC, Goncharov T, Newton K, Vucic D (2015). Cellular IAP proteins and LUBAC differentially regulate necrosome-associated RIP1 ubiquitination. Cell Death Dis.

[102] Wang H, Meng H, Li X, Zhu K, Dong K, Mookhtiar AK (2017). PELI1 functions as a dual modulator of necroptosis and apoptosis by regulating ubiquitination of RIPK1 and mRNA levels of c-FLIP. Proc Natl Acad Sci USA.

[103] Douglas T, Saleh M (2019). Post-translational modification of OTULIN regulates ubiquitin dynamics and cell death. Cell Rep.

[104] Li X, Zhang M, Huang X, Liang W, Li G, Lu X (2020). Ubiquitination of RIPK1 regulates its activation mediated by TNFR1 and TLRs signaling in distinct manners. Nat Commun.

[105] Onizawa M, Oshima S, Schulze-Topphoff U, Oses-Prieto JA, Lu T, Tavares R (2015). The ubiquitin-modifying enzyme A20 restricts ubiquitination of the kinase RIPK3 and protects cells from necroptosis. Nat Immunol.

[106] Roedig J, Kowald L, Juretschke T, Karlowitz R, Ahangarian Abhari B, Roedig H, et al. USP22 controls necroptosis by regulating receptor‐interacting protein kinase 3 ubiquitination. EMBO Rep. 2020. 10.15252/embr.202050163. 10.15252/embr.202050163 PMC7857539 33369872

[107] Lee SB, Kim JJ, Han SA, Fan Y, Guo LS, Aziz K (2019). The AMPK–Parkin axis negatively regulates necroptosis and tumorigenesis by inhibiting the necrosome. Nat Cell Biol.

[108] Seo J, Lee EW, Sung H, Seong D, Dondelinger Y, Shin J (2016). CHIP controls necroptosis through ubiquitylation-and lysosome-dependent degradation of RIPK3. Nat Cell Biol.

[109] Mei P, Xie F, Pan J, Wang S, Gao W, Ge R (2021). E3 ligase TRIM25 ubiquitinates RIP3 to inhibit TNF induced cell necrosis. Cell Death Differ.

[110] Choi SW, Park HH, Kim S, Chung JM, Noh HJ, Kim SK (2018). PELI1 selectively targets kinase-active RIP3 for ubiquitylation-dependent proteasomal degradation. Mol Cell.

[111] Petersen SL, Chen TT, Lawrence DA, Marsters SA, Gonzalvez F, Ashkenazi A (2015). TRAF2 is a biologically important necroptosis suppressor. Cell Death Differ.

[112] O’Donnell MA, Perez-Jimenez E, Oberst A, Ng A, Massoumi R, Xavier R (2011). Caspase 8 inhibits programmed necrosis by processing CYLD. Nat Cell Biol.

[113] Moquin DM, McQuade T, Chan FKM (2013). CYLD deubiquitinates RIP1 in the TNFα-induced necrosome to facilitate kinase activation and programmed necrosis. PLoS ONE.

[114] Dondelinger Y, Declercq W, Montessuit S, Roelandt R, Goncalves A, Bruggeman I (2014). MLKL compromises plasma membrane integrity by binding to phosphatidylinositol phosphates. Cell Rep.

[115] Murphy JM, Czabotar PE, Hildebrand JM, Lucet IS, Zhang JG, Alvarez-Diaz S (2013). The pseudokinase MLKL mediates necroptosis via a molecular switch mechanism. Immunity.

[116] Cai Z, Jitkaew S, Zhao J, Chiang HC, Choksi S, Liu J (2014). Plasma membrane translocation of trimerized MLKL protein is required for TNF-induced necroptosis. Nat Cell Biol.

[117] Chen X, Li WW, Ren J, Huang D, He WT, Song Y (2014). Translocation of mixed lineage kinase domain-like protein to plasma membrane leads to necrotic cell death. Cell Res.

[118] Wang H, Sun L, Su L, Rizo J, Liu L, Wang LF (2014). Mixed lineage kinase domain-like protein MLKL causes necrotic membrane disruption upon phosphorylation by RIP3. Mol Cell.

[119] Garcia LR, Tenev T, Newman R, Haich RO, Liccardi G, John SW (2021). Ubiquitylation of MLKL at lysine 219 positively regulates necroptosis-induced tissue injury and pathogen clearance. Nat Commun.

[120] Liu Z, Dagley LF, Shield-Artin K, Young SN, Wang X, Tang M, et al. Oligomerization-driven MLKL ubiquitylation antagonises necroptosis. bioRxiv. 2021. 10.15252/embj.2019103718. 10.15252/embj.2019103718 PMC8634140 34698396

[121] Holohan C, Van Schaeybroeck S, Longley DB, Johnston PG (2013). Cancer drug resistance: an evolving paradigm. Nat Rev Cancer.

[122] Moujalled D, Strasser A, Liddell JR (2021). Molecular mechanisms of cell death in neurological diseases. Cell Death Differ.

[123] Humphreys LM, Smith P, Chen Z, Fouad S, D’Angiolella V (2021). The role of E3 ubiquitin ligases in the development and progression of glioblastoma. Cell Death Differ.

[124] Walczak H (2011). TNF and ubiquitin at the crossroads of gene activation, cell death, inflammation, and cancer. Immunological Rev.

[125] Kist M, Kőműves LG, Goncharov T, Dugger DL, Yu C, Roose-Girma M, et al. Impaired RIPK1 ubiquitination sensitizes mice to TNF toxicity and inflammatory cell death. Cell Death Differ. 2020;28:985–1000. 10.1038/s41418-020-00629-3 PMC7937686 32999468

[126] Tang Y, Tu H, Zhang J, Zhao X, Wang Y, Qin J, et al. K63-linked ubiquitination regulates RIPK1 kinase activity to prevent cell death during embryogenesis and inflammation. Nat Commun. 2019; 10. 10.1038/s41467-019-12033-8. 10.1038/s41467-019-12033-8 PMC6744441 31519887

